# Pathogenesis, Diagnosis and Management of Squamous Cell Carcinoma and Pseudoepithelial Hyperplasia Secondary to Red Ink Tattoo: A Case Series and Review

**DOI:** 10.3390/jcm12062424

**Published:** 2023-03-21

**Authors:** Yasmina Rahbarinejad, Pedro Guio-Aguilar, Anh Ngoc Vu, Michael Lo, Christine McTigue, Alex Nirenberg, Warren M. Rozen

**Affiliations:** 1 College of Medicine and Dentistry, James Cook University, Townsville, QLD 4811, Australia; 2 Plastic and Reconstructive Surgery, Peninsula Health, Melbourne, VIC 3199, Australia; 3 Plastic and Reconstructive Surgery, Monash Health, Melbourne, VIC 3168, Australia; 4 Dorevitch Pathology at Peninsula Health, Melbourne, VIC 3199, Australia

**Keywords:** pseudoepitheliomatous hyperplasia, squamous cell carcinoma, red, tattooing, diagnosis, treatment

## Abstract

The increasing popularity of tattooing has paralleled an increase in associated cutaneous reactions. Red ink is notorious for eliciting cutaneous reactions. A common reaction is pseudoepitheliomatous hyperplasia (PEH), which is a benign condition closely simulating squamous cell carcinoma (SCC). Differentiating PEH from SCC is challenging for pathologists and clinicians alike. The exact pathogenesis of these lesions secondary to red ink is not known, and there are no sources outlining diagnostic and treatment options and their efficacy. We present four study cases with different pathologies associated to red ink tattoos including lichenoid reaction, granulomatous reaction, PEH, and an SCC. Additionally, an extensive review of 63 articles was performed to investigate pathogenesis, diagnostic approaches, and treatment options. Hypotheses surrounding pathogenesis include but are not limited to the carcinogenic components of pigments, their reaction with UV and the traumatic process of tattooing. Pathogenesis seems to be multifactorial. Full-thickness biopsies with follow-up is the recommended diagnostic approach. There is no evidence of a single universally successful treatment for PEH. Low-dose steroids are usually tried following a step up in lack of clinical response. For SCC lesions, full surgical excision is widely used. A focus on clinicians’ awareness of adverse reactions is key for prevention. Regulation of the unmonitored tattoo industry remains an ongoing problem.

## 1. Introduction

Squamous cell carcinoma (SCC) is the second most common form of skin cancer, which is characterised by the abnormal, locally invasive growth of squamous cells [1]. Pseudoepitheliomatous hyperplasia (PEH) is a benign hyperproliferation of the epidermis and adnexal epithelium closely simulating SCC both clinically and on pathologic examination [2,3]. While not considered a neoplasm, PEH is a reactive histological pattern to various stimuli [2].

Tattooing may elicit hypersensitivity, infectious, neoplastic, isomorphic and granulomatous complications which may be cutaneous or systemic, acute or chronic, delayed or acute, benign or malignant [3]. The growth in popularity of tattooing in recent decades has paralleled with an increase in the number of adverse effects associated with tattooing [4]. In the literature, PEH and SCCs are reported as rare complications of tattoos, with most cases reporting localisation to the red ink portion of tattoos. Despite the rising popularity of tattooing, only a handful of these cases have been reported. Distinguishing between SCC and PEH can be challenging for the pathologists and clinicians [2]. In order to easily rule out the possibility of a disguised neoplastic process or allow for its timely treatment, clinicians need to be aware of such presentations. Current data on diagnostic approaches and treatment modalities are scarce and limited to individual case reports. This study aims to investigate the spectrum of presentations, pathogenesis, and current diagnostic and treatment approaches reported in the literature to help guide appropriate management.

## 2. Materials and Methods

This study aims to investigate the spectrum of presentations of squamous change associated with red tattoo ink by reporting on cases of cutaneous adverse reactions and their management coupled with a review of the literature on pathogenesis, current diagnostic, and treatment approaches to help guide appropriate management.

This study comprises a case series and review of the literature. The cases represent clinical cases with histopathological diagnosis of either SCC or PEH. The diagnostic approaches, management plans, progress and outcome of these cases were recorded and compared. Secondly, an OVID MEDLINE literature search using the MeSH terms “Pseudoepitheliomatous Hyperplasia” or “Carcinoma Squamous Cell” or “Lichenoid Eruptions” or “Lichen Planus” or “Pseudolymphoma” AND “Tattooing” conducted on 6 April 2022. This search yielded 114 results. Studies published from 1946 to April 2022 available in the English language with no restriction on the study design were included. In total, 100 articles remained after imposing these restrictions. After reviewing the title and abstract, a further 37 articles were excluded due to focusing on other cutaneous manifestations of tattooing such as lymphomas. Case reports were excluded if too little information was available to confirm a diagnosis of SCC or PEH or no clinical characteristics regarding the patient, investigations or course of reaction were available. The literature focusing on lesions secondary to red ink were prioritised followed by lesions secondary to other coloured inks and finally the literature available on kerato-acanthoma (KA) secondary to red ink was used to compare.

## 3. Results

The case series comprises of two cases of PEH, a case of SCC and a case of granulomatous reaction and their respective investigations, histological findings, treatment, and outcome of treatment.

### 3.1. Case Reports

#### 3.1.1. Case 1

**Figure 1:**
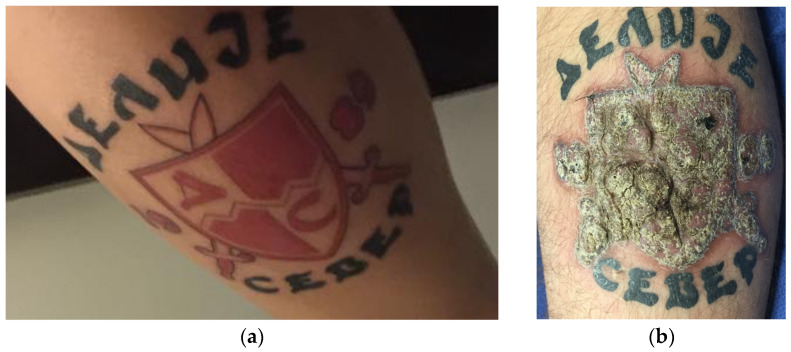
Photograph taken immediately after tattoo was imprinted (**a**). Photograph taken six months after receiving the tattoo. Note the scaly, raised exophytic lesion in areas tattooed with red ink only while the black ink areas remain spared (**b**).

**Figure 2:**
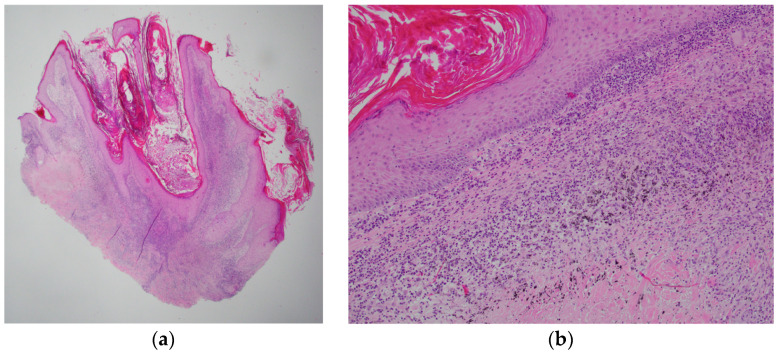
Skin biopsy at 12.5× magnification (**a**) showing orthokeratosis, hyperplastic squamous epithelium, papillomatous change and lichenoid inflammation at superficial dermis. At 100× magnification (**b**), tattoo pigment and lichenoid reaction is shown in the superficial dermis.

A thirty-year-old male, smoker, an otherwise healthy male, was referred to the Plastic Surgery clinic with a local reaction which started six months after procuring a tattoo on his lower leg from a tattoo shop in Europe. The patient described progressive swelling, blistering and pruritus limited to the areas of red ink over several weeks. The lesion was painless. The red-inked tattooed area became a raised, scaly, exophytic plaque. Notably, the areas of black ink remained unaffected (Figure 1a,b). A skin biopsy of the affected area showed focal lichenoid cutaneous reaction changes (Figure 2a,b).

This patient was treated with injection of steroid (1.5 mls of triamcinolone acetonide 40) throughout the lesion followed by the application of topical betamethasone three times daily for a month. Four weeks later, there was clinical improvement with reduction in pruritus, scaling and swelling of the lesion.

Three months post-treatment, however, he reported further progression of the scaling and pruritis. Shave biopsy revealed a foreign body reaction to the tattoo pigment with pseudoepitheliomatous hyperplasia of the epidermis. Histiocytes and multi-nucleated giant cells were noted focally with small granuloma formation consistent with a granulomatous-type reaction to red ink cutaneous tattooing. Microbiological testing was negative on bacterial culture, staining for acid fast bacilli and fungi by Ziehl-Neelsen and Grocott methods, respectively, were negative, and mycobacterium polymerase chain re-action testing was also negative.

**Figure 3:**
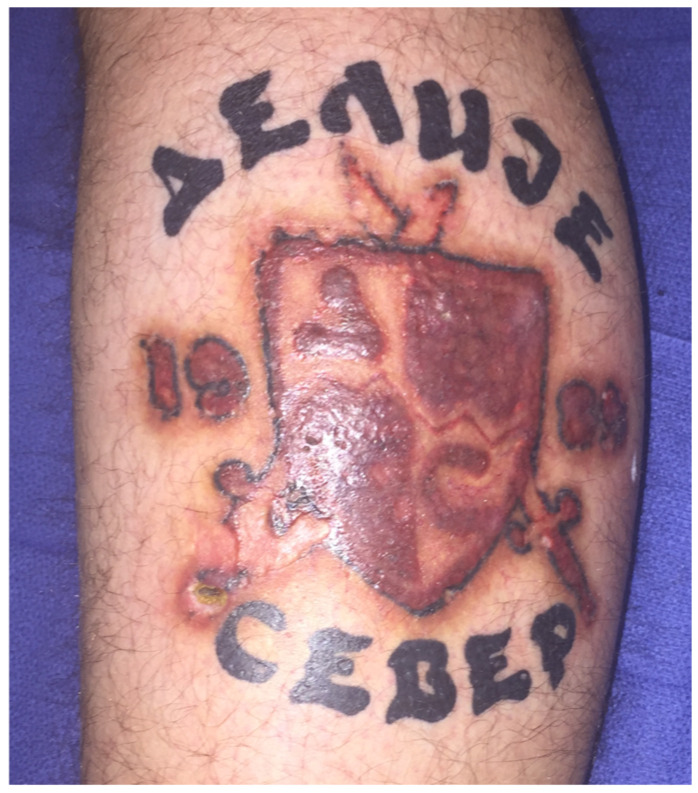
Eighteen months follow up with significant clinical improvement after management with six sessions of intra-lesional steroid injections and topical corticosteroid. The tattoo is no longer raised, scaly or pruritic. The tattoo is largely preserved with an acceptable aesthetic outcome.

Due to further progression of symptoms, intra-lesional injection of triamcinolone acetonide 40 was repeated six months after the initial intervention, the patient receiving intra-lesional injections of triamcinolone acetonide 40 at monthly intervals for six months. He eventually showed slow but progressive reduction in symptoms. Eighteen months after presentation, the patient had significant clinical improvement and was satisfied with the aesthetic outcome, allowing preservation of his tattoo (Figure 3).

#### 3.1.2. Case 2

**Figure 4:**
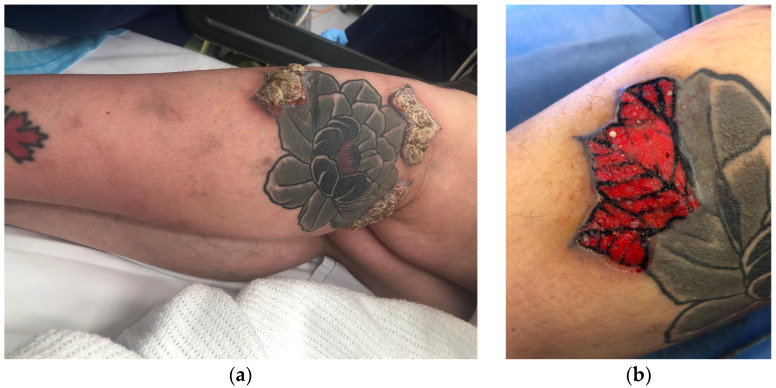
Skin lesion affecting exclusively red-ink tattooed areas 6 months after initial tattoo was completed (**a**). Macroscopic appearance after deep shave biopsy was performed (**b**).

A fifty-three-year-old female presented to her general practitioner (GP) with skin changes and inflammation on her left leg tattoos six months after she had tattooing artwork completed in the United States. Punch biopsies taken by her general practitioner showed inflammation and atypical squamous proliferation suspicious for SCC. She was referred to plastic surgery. Clinically, there were raised keratotic lesions with surrounding erythema limited to skin tattooed in red ink in three separate areas of the proximal leg, sparing the rest of the skin tattooed with black ink (Figure 4a,b). There were less severe skin changes, following the same pattern, in tattooed areas of the mid leg and foot which were tattooed at the same time.

**Figure 5:**
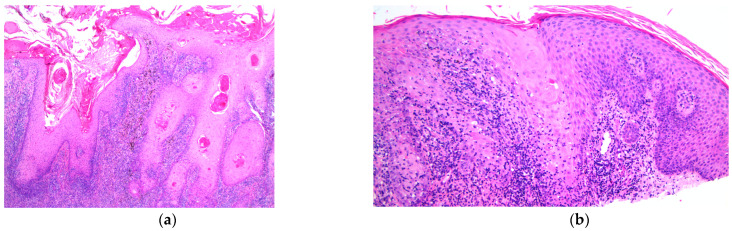
Histopathological photograph at 40× magnification (**a**) and 100× magnification (**b**) showing the features of the pseudoepitheliomatous hyperplasia (PEH) with haematoxylin eosin stain (H&E).

Deep shave biopsies were taken from all four separate lesions and the case presented at the skin cancer multidisciplinary team meeting (Figure 3). Tissue bacterial and fungal cultures showed no growth. PCR for Mycobacterium ulcerans was negative. Histopathology findings were in keeping with a lichenoid type inflammatory reaction with pseudoepitheliomatous hyperplasia (Figure 5a,b). The patient is having clinical monitoring every three months.

#### 3.1.3. Case 3

**Figure 6:**
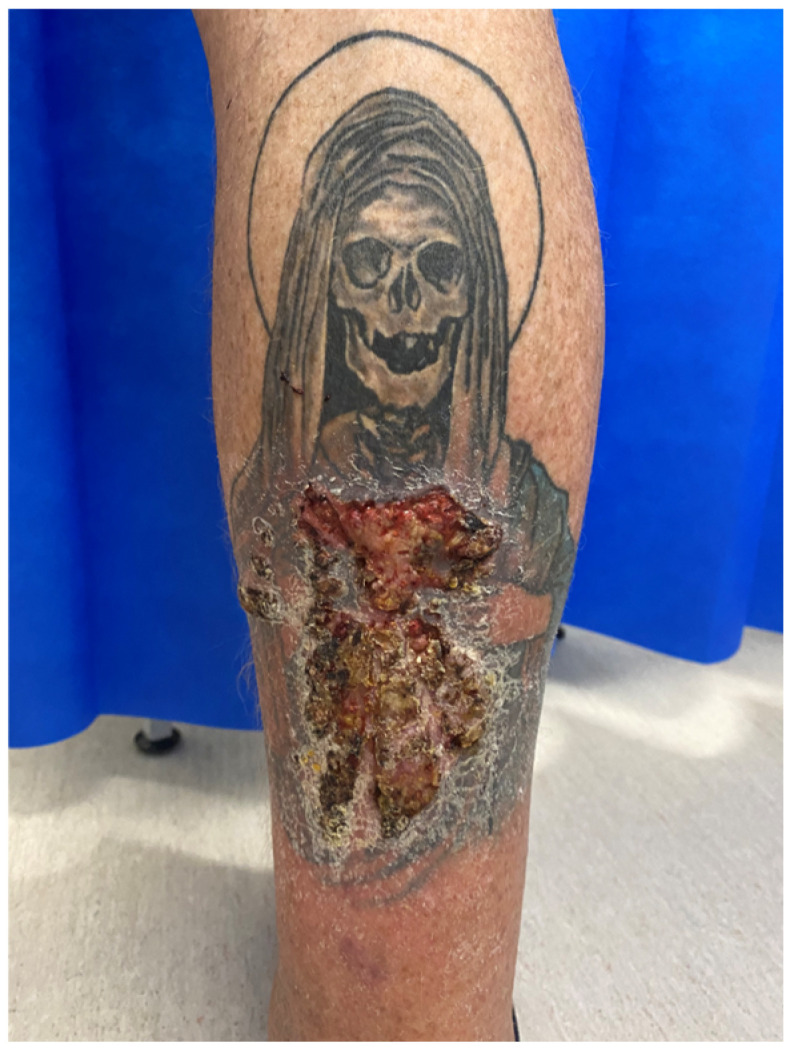
Macroscopic appearance after deep shave biopsy was performed.

**Figure 7:**
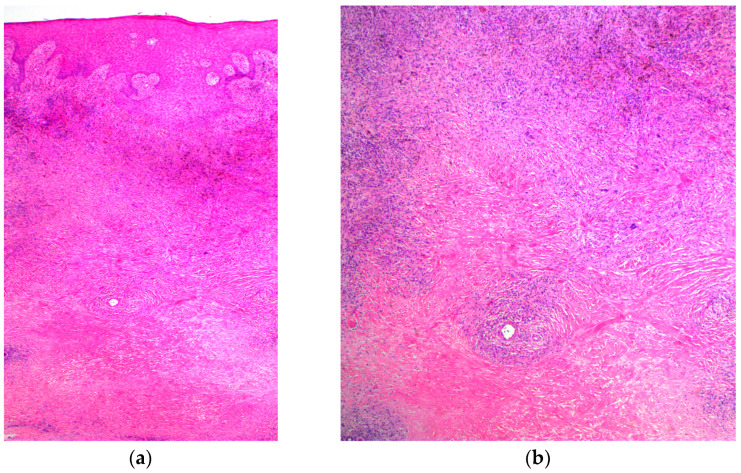
Histological features of necrotising granulomatous reaction. Note the necrosis in the dermis, surrounding giant cells and some pigment. (**a**) low power; (**b**) high power.

A fifty-two-year-old man presented to the plastic surgery clinic with a non-healing ulcer overlying the red-inked part of a skin tattoo that was completed five years earlier (Figure 6). Punch biopsies demonstrated epidermal hyperplasia and necrotising granulomatous inflammatory changes with histiocytes, multi-nucleated giant cells and a lymphocytic infiltrate extending into the dermis (Figure 7a,b). M. Ulcerans PCR and bacterial and fungal cultures were negative.

#### 3.1.4. Case 4

**Figure 8:**
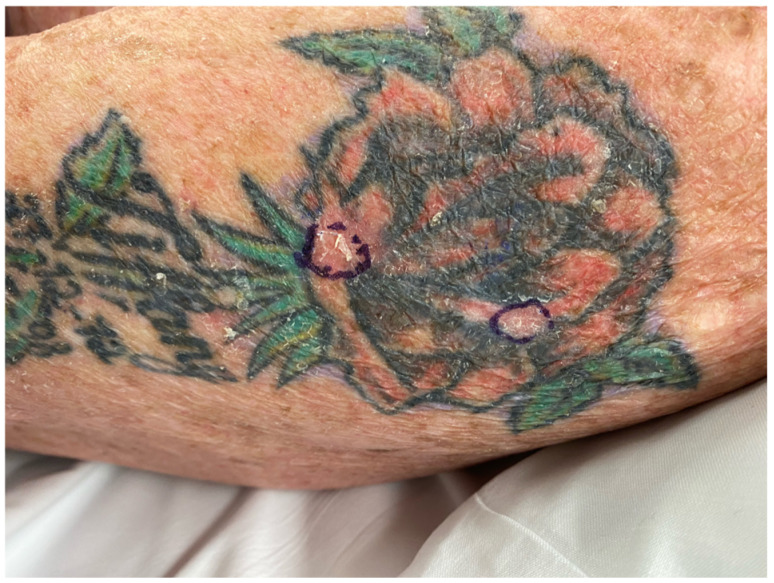
Clinical photograph of two scaly lesions of the skin located in the red-inked areas of a multi-coloured tattoo of the left arm completed 13 years earlier.

A fifty-two-year-old male presented with two cutaneous lesions within a tattoo of his left arm 13 years after having their tattoo appointment. Tattoo had three pigments—black, red and blue; however, lesions were confined solely within the red tattoo ink (Figure 8). Patient was a smoker, had no history of skin cancers and no other relevant past medical history. Shave biopsy revealed solar keratosis along with a well-differentiated invasive SCC. The epidermis shows full-thickness dysplasia, acanthosis, hyperkeratosis and parakeratosis, on a background of lichen simplex chronicus. The superficial dermis showed a chronic inflammatory infiltrate. This lesion was managed with formal excision with clear margins and healed well with no complications.

## 4. Discussion

### 4.1. Pathogenesis of Cutaneous Reactions Secondary to Red Ink Tattoo

The exact pathogenesis of SCC or PEH secondary to tattoo is not agreed upon in the literature. Some suggest that a malignant or a premalignant lesion can also be tattooed over and mask its evolution, especially since SCC is the second-most prevalent cancer of the skin [5]. However, rapidly evolving lesions localised to the red part of tattoo, most arising within 1 week to 1 year, with 2 years being the longest reported onset for PEH, contradict fortuitous aetiology [2]. This argument does not prove a non-fortuitous aetiology for SCCs as there are reports of SCCs arising in very old tattoos (McQuarrie et al. reports incidence in a 21-year-old red ink tattoo; Sarma et al. in a 50-year-old black tattoo) [6,7]. McQuarrie et al. fails to demonstrate delay in onset as the time interval from first occurrence of a lesion to cancer diagnosis is not reported [6]. The first occurrence of skin irritation in Sarma et al. is reported to be recent to diagnosis; however, this provides no information on the onset of the SCC itself [7]. PEH’s low incidence, presentation among the young and healthy, coupled with incidence secondary to infections, neoplasia, inflammation and trauma suggest that incidence cannot be purely casual [3,7,8,9].

Tattooing is a traumatising process characterised by puncturing of the dermis and the introduction of exogenous materials. Trauma may be a specific triggering factor as most cases of SCC and PEH arise within the first year after tattooing [7]. PEH has also reported post Mohs micrographic surgery, reaffirming a potential role for trauma [10]. PEH is associated with clinical injuries, such as chronic dermopathies, lymphedema, cutaneous infections and infestations [11]. PEH is said to be a response to infectious, inflammatory and neoplastic conditions such as chronic osteomyelitis or cutaneous lymphoma [9]. Other proposed aetiologies include an isomorphic response of other skin pathologies such as psoriasis or lichen planus; however, this is observed in all tattooing nonspecific to the ink colour [2,12]. Others propose the hyperplasia of PEH to be leukocytes induced and primarily an autoimmune condition or an unusual and severe manifestation of allergic contact dermatitis [13]. Foreign body reactions, TNF alpha, ROS induction, phototoxicity, and photochemistry are all cofactors in the allergic induced pathogenesis of reactive lesions such as PEH [8]. The chronic inflammation post-tattoo placement is thought to be more strongly associated with development of SCC due to later onset of development compared to PEH [14]. However, trauma alone cannot explain the rise of SCC or other cutaneous manifestations within an individual pigment colour [7]. If the trauma, scarring process or chronic inflammation play such an important role, we would expect a much higher incidence of SCC and PEH in all traumatic processes and not only tattoos.

Red pigments are the most reported cause of cutaneous reaction to tattoos [2]. Cinnabar red, the pigment containing mercury sulphate and a well-known allergen implicated in delayed hypersensitivity reactions, is reported to be the cause in presentations of PEH secondary to red [2,13,15,16,17]. Recently, inorganic dyes in red tattoos such as cinnabar have been replaced by organic ones, such as azo pigments, quinacridone and polycyclic-compounds; however, this has not reduced the presentation of PEH or SCC secondary to red ink [2]. After black, red is the next most popular ink in tattoos [18]. This might explain the over-representation of SCC or PEH in red ink; however, this is contradicted by localisation to the red area of the tattoos [11].

A survey of tattooists in Australia coupled with chemical analysis of commonly used inks by NICNAS in 2014–2015 revealed that the composition of red pigments is varied, often including ingredients not reported on the labels (often non-intentional breakdown products or contaminants) [19]. The report also revealed the use of inks not intended for tattooing in Australia [19]. Lack of data on the exact ingredients and in vivo studies makes it difficult to determine the exact entity implicated in pathogenesis of PEH and SCC secondary to red tattoos. Both SCC and PEH are reported in other ink pigments. Balfour et al. reported a case of PEH occurring secondary to manganese-based purple pigment [20]. Pitarch et al. and Sarma et al. sport SCCs localised to black ink [6,21]. Paprottka et al. report a case of SCC in dark blue pigment [22]. These cases compromise the role of red ink pigment in the pathogenesis of such lesions.

The localisation of most cases into tattoos in the distal limbs, areas that are regularly exposed to sunlight, suggest a role for UV in pathogenesis [2]. Substances such as 2- anisidine form toxic or carcinogenic products such as 3, 3-dichlorobenzidine in combination with sunlight exposure [2,23]. In vitro studies have shown that solar radiation has the potential to increase the decomposition of pigment into hazardous aromatic amines or to generate cytotoxic singlet oxygen [23]. Pigment Red 22 has been identified as an ingredient of tattoo inks in Australia [19]. Dithionite reduction in Pigment Red 22 produces 2,4- toluenediamine, which is a highly toxic compound [19]. Cadmium sulphide, a component in some red inks, is known to have phototoxic qualities which are associated with inflammation [5].

There is strong evidence for implication of UV in pathogenesis of such lesions; however, UV is not the sole culprit in all cases of PEH and SCC secondary to red ink tattoo. The literature provides little information on the sun habits of individuals with SCC of PEH secondary to red tattoos. Cases that provide information on sun habits deny prolonged sun exposure or sunburns. Additionally, tattooed individuals avoid sun exposure to protect the pigment of their tattoos.

### 4.2. Common Histopathological Features

Tattoo reactions can be broadly classified into allergic hypersensitivity, acute inflammatory, granulomatous, lichenoid and pseudolymphomatous-type reactions [24,25]. Granulomatous and lichenoid hypersensitivity occurs less frequently than eczematous reactions. Granulomatous reactions often occur months to years after the tattoo was initially acquired, such as in Case 1. The clinical presentation is similar, with evolving pruritus, localised oedema, and an eczematous eruption [26]. The type of reaction is classified based on histological features. Granulomatous reactions typically show epithelioid cells and lymphocytes in addition to the characteristic multi-nucleated giant cells, whereas lichenoid reactions show a predominantly T-cell infiltrate which causes basal layer damage [26,27]. While epicutaneous patch testing can be used in the diagnosis of eczematous reactions, it has no value in the diagnosis of granulomatous and lichenoid reactions.

The histopathologic features of pseudoepitheliomatous hyperplasia (PEH) include florid amhyperplasia of the epidermis and marked eosinophilia in some areas. There is marked elongation of the rete ridges as well as prominence and hyperplasia of follicular epithelium. Inflammatory cells infiltrate scarred dermis, which contains tattoo pigment. The inflammatory infiltrate is predominantly lymphohistiocytic in type but also contains scattered plasma cells and eosinophils extending to the dermoepidermal junction. There is a notable exocytosis of inflammatory cells into both the hyperplastic epidermis and the follicular epithelium. The main differential diagnosis is well-differentiated SCC. The presence of squamous proliferation in areas of pseudoepitheliomatous hyperplasia (PEH) makes the entities difficult to differentiate histopathologically, especially in shallow biopsies (Figure 8).

The lichenoid type of reaction pattern can be similar in appearance to hypertrophic lichen planus in the histopathological images. It has been suggested that these reactions might best be referred to as lichenoid reaction with pseudoepitheliomatous hyperplasia or a hypertrophic lichen planus (HLP)-like reaction [28]. The recognition of an inflammatory component may allow additional treatment options.

Cutaneous pseudolymphoma (CSL) is an unusual immune response that can be caused by red ink tattoos. This diagnosis can be confirmed by histopathology, immunohistochemistry, and the polymerase chain reaction testing of tissue, which will show a polyclonal lymphoid infiltrate with a lichenoid reaction at the junction [29]. CPL is very rare, and it is benign, but as it may evolve into a true lymphoma, close follow up is recommended [30].

These changes are all able to be differentiated on histopathology, from PEH and SCC, and complete histologic assessment is thus paramount. The diagnostic approach to this is described below.

### 4.3. Diagnostic Approaches for PEH and SCC

Distinguishing PEH and SCC can be challenging. Formal histological biopsy is the main diagnostic tool. This can be due to a similar pattern of exuberant epidermal proliferation, lack of consensus on diagnostic criteria and selection of biopsy site and type [11]. Guidelines suggest a complete excisional biopsy including the base of the lesion and underlying dermis to differentiate SCC from other similar lesions such a keratoacanthoma, as the depth of the lesion, invasion through the basement membrane, and non-uniform changes can mean that diagnostic features are missed in limited or shallow biopsies [2,31]. Partial biopsy will generally be unhelpful and almost always be reported as SCC, as the architecture of the entire lesion is required to evaluate the possibility of PEH or keratoacanthoma [31]. KA and PEH both have adnexal origin and infundibular hyperplasia, glassy keratinocytes cytoplasm and crateriform architecture; thus, distinguishing between them can be challenging, especially in case of superficial biopsy. For instance, Tammaro et al. diagnosed a case of PEH through punch biopsies [13]. Kheradmand et al. availed two 6 mm punch biopsies for diagnosis of PEH secondary to purple tattoo [32]. Cipollaro diagnosed KA only on a biopsy performed by shaving [33]. Lack of evaluation of the entirety of lesions raises doubt on the real nature of these lesions.

Full-thickness biopsies and/or surgical removal for histological examination followed by long-term follow-up must be performed to exclude SCC as a potential diagnosis. PEH is not fundamentally a hyperplasia of epidermal epithelium but rather a hyperplasia of adnexal epithelia, namely of follicular infundibula and eccrine ducts closely simulating SCC. PEH shows irregular invasion of the dermis by uneven, jagged, often pointed epidermal cell masses and strands with horn-pearl formation. An irregular proliferation of epidermis may extend below the level of the sweat gland, where they appear as isolated islands of epidermal tissue. Furthermore, the lymphocytic invasion of epithelium and disintegration of some of the epidermal cells seen in PEH are absent in SCC. Verrucous carcinoma shows a verrucous upward and downward proliferation with more pronounced keratinisation in downward extension, which appears bulbous rather than sharply pointed as in PEH [34]. Due to the complexities involved in differentiating this condition, all histological findings should be reported to clinicians regardless of nomenclature in order to guide appropriate management and close follow-up [3]. p53 immunostaining may be of help in this setting. PEH generally has less intense and extensive staining compared with that of SCC [35]. The genes C15orf48 and KRT9 had a distinct and robust gene expression pattern in distinguishing squamous cell carcinoma from pseudoepitheliomatous hyperplasia [36]. C15orf48 had higher expression than KRT9 in squamous cell carcinoma but lower expression than KRT9 in pseudoepitheliomatous hyperplasia [36]. A multiplex TaqMan PCR assay may be used as a helpful ancillary molecular diagnostic test to accurately distinguish squamous cell carcinoma from pseudoepitheliomatous hyperplasia in challenging cases [36].

Most of the diagnostic approaches in the literature focus on histological differences. The onset of the presentation after the tattooing procedure is a clinical factor that can differentiate between SCC and PEH, but it is often overlooked. PEH can occur rapidly after tattooing [5,20,21]. Between the cases reviewed, the longest reported interval between onset of PEH and tattoo procedure was 2 years [2]. In contrast, SCC can present 50 years post-tattoo [7]. Attention to clinical details coupled with histological evaluation can minimise the chance of diagnostic errors and optimise treatment and follow-up.

Tattoos are associated with transmittable diseases such as leprosy and syphilis. Consideration of these differentials is essential in the management of tattoo-related reactions. Patch tests (SIDAPA and FIRMA special series) have been conducted in certain cases; however, findings were insignificant and did not guide management [8,13]. Chronic infections with bacteria, fungi, parasites, and viruses have been shown to have a role in the pathogenesis of SCC [37]. PEH occurs in the setting of cutaneous infections and has been reported with many types of protozoans, viral, bacterial, mycobacterial, and fungal infections and infestation [11]. To address this, special stains may be applied to exclude the presence of fungal, bacterial, or mycobacterial organisms. Periodic acid–Schiff, acid-fast, and Grocott’s methenamine silver have been applied in case 1 and mycobacteria PCR, bacterial and fungal staining applied in all the cases mentioned; both had insignificant findings. PEH-associated granulomatous lesions in the dermis have been reported [38]. It is important to distinguish such reactive changes from systemic diseases, such as sarcoidosis and tuberculosis.

### 4.4. Efficacious Treatment

Currently, there is no evidence in the literature to support a single universally successful treatment for PEH. Intervention options range widely and include antihistamines, oral and topical steroid use, ablation with laser and surgical excision [39]. Steroids are usually tried in the first instance, aid in symptomatic relief and can be administered orally or intra-lesionally. The topical application of corticosteroid does not, however, allow penetration into subdermal tissue. Less aggressive steroids are administered initially to avoid adverse effects. This is then followed by stronger formulations if unresponsive. A case of PEH secondary to purple ink was treated with of Clobetasol 0.05% ointment under occlusion for 1 month with no improvement [32]. This prompted a change to intralesional triamcinolone 5 mg/mL and tacrolimus, which resulted in both clinical symptomatic relief and atrophy of the plaque [32]. Kluger et al. reported the complete subsidence of a PEH nodule after Clobetasol 0.05% ointment use for a month [40]. However, this corresponded with pain and skin atrophy, which prompted the protracted application of such potent corticosteroids [40]. PEH may persist for months or years despite topical, intralesional, or systemic steroid therapy. Laser therapy has been suggested when conservative management with steroid fails [39]. Laser therapy can be achieved with CO2 laser or Q-switched Nd:Yag laser. [41]. It requires multiple sessions and can be expensive [41]. The administration of topical 5-FU or CO2 laser reveal varying therapeutic efficacy in the management of PEH [2]. The histological similarity of PEH with hypertrophic lichen planus has encouraged the use of photochemotherapy (PUVA), phototherapy (narrowband UVB), excimer laser or photodynamic therapy, and topical calcineurin inhibitors [2,42]. Reports of these treatment modalities is scarce and often anecdotal [2,42]. Further interventions include cryotherapy, electrosurgery and dermabrasion [26]. Responses to treatment are often variable and are influenced by the severity and extent of the reaction. Surgical excision, with or without skin grafts, should be considered when all other interventions have failed [39,43]. However, it is difficult to establish the depth of the inflammatory reaction into surrounding tissues prior to surgery. This means that subdermal surgical excision may provide a suboptimal aesthetic outcome [44].

Review of the literature demonstrated that complete surgical excision is the most common method of treatment for SCC [6,21,45]. The size of lesions guides the area to be excised. Sherif et al. reported a case of SCC that required a wide local excision and closing with a split-thickness skin graft [14]. Paprottka et al. report performing a second excision and closing with split-skin graft following further skin alterations on the red parts of the multi-coloured tattoo [22]. Ortiz and Yamauchi report using Moh’s microsurgery on lips and Tan-Billet et al. report using it on facial areas where tissue conservation is considered critical [14].

## 5. Conclusions

The exact pathogenesis of SCC and PEH secondary to red ink tattoos remains unclear. The long-term follow-up of red ink tattoos would provide more information around the pathogenesis and will reveal if red ink is an independent risk factor or only a coincidental association. Clinicians should be aware of this entity and continue reporting such cases. These reactions are expected to be increasingly encountered in trends such as permanent cosmetic make-up. Clinician awareness will aid in the timely treatment of neoplastic processes. Studies on contents of cutaneous ink and their by-products are needed in order to develop standardised regulations around ink content and use in Australia.

## Data Availability

The data presented in this study are openly available in Ovid MEDLINE.
